# Quality of Life of People with Cancer in the Era of the COVID-19 Pandemic in India: A Systematic Review

**DOI:** 10.2174/1745017902117010280

**Published:** 2021-12-31

**Authors:** Kusum K. Rohilla, C Vasantha Kalyani, Sweety Gupta, Amit Gupta, Manoj Gupta

**Affiliations:** 1 College of Nursing, All India Institute of Medical Sciences, Rishikesh, India; 2 Radiation Oncology, All India Institute of Medical Sciences, Rishikesh, India; 3 Department of Surgery, All India Institute of Medical Sciences, Rishikesh, India

**Keywords:** Quality of life, Cancer, COVID-19, Systematic review, Chemotherapy, Palliative care

## Abstract

**Background:**

The recent pandemic of COVID-19 caused havoc on the health system globally and raised a lot of questions and issues. Treatment for cancer is an emergency that cannot be taken back, particularly in an era of global pandemics. Cancer treatment mainly includes chemotherapy, surgery, radiotherapy, and palliative care, and because of the pandemic, all of these treatments are affected. The COVID-19 pandemic also had a potential effect on the quality of life and mental health of patients as well as health workers.

**Objective:**

This systematic review was intended to discuss the quality of life of people with cancer in the era of the COVID-19 pandemic in India in the light of the best available facts.

**Methods:**

An extensive literature search was done on PubMed, Medline, Embase, Clinical Key and Google Scholar databases till 3rd Feb 2021. Out of 1455 research articles, 06 research articles were included in this systematic review.

**Results:**

The results showed that cancer treatment delivery was as per standard safety protocol and the best treatment decisions were made by scheduling and setting priority. Till data, no direct research was conducted on the Indian continent to assess the quality of life of cancer patients in the COVID-19 era. The effect on the quality of life of cancer patients is very large and needs to be explored more by further research. Issues to be discussed with health care administrators and policy makers further. The tele-oncology method of cancer care delivery to patients is another rational option which is applicable as well.

**Conclusion:**

This systematic review demonstrated up-to-date evidence regarding the quality of life of cancer patients in the COVID-19 era in India. No research has been done to assess the quality of life of cancer patients. Still, the area is unrevealed, but evidence from other global studies indicates an altered quality of life for cancer patients. To maintain quality of life, cancer physicians should make evidence-based decisions and incorporate multidisciplinary management into decision making.

## 1 INTRODUCTION

Globally, the year 2020 was challenging due to the COVID-19 pandemic [1], and still, in the year 2021, we are not in a better situation to predict the end of this problem [2]. The health system mainly targets preventing contamination and transmission of this deadly virus and treating infected patients [3]. One can never imagine the effects of COVID-19 on a person who is already suffering from chronic diseases like cancer [4]. Worldwide, due to the COVID-19 pandemic, there has been a significant decrease in cancer treatment [5]. Global data also indicated a substantial decline in cancer diagnosis, screening, and treatment, including chemotherapy, radiation therapy, surgery, and palliative care. Research evidence shows a delay in treatment of cancer for two months (due to diagnostic or referral reasons), which leads to a loss of 0 to 7 years of life for one cancer patient [6].

The four main treatment modalities, *i.e*., hemato-oncology, radiation oncology, surgical oncology, and palliative care, are provided to any cancer patient [7]. As a global pandemic, we should appreciate the efficacy of these modalities for cancer treatment [8]. For cancer and its treatment, time is the most important dimension in deciding the prognosis of any patient. Cancer treatment cannot wait until COVID-19 has taken a back seat [9]. The main property of cancer treatment is its prompt intervention, which is greatly influenced by the existing condition of healthcare facilities around us and is more than overwhelmed by the COVID-19 pandemic [10]. The main challenge which was faced during this time was the delivery of oncology care, where patients faced problems with the accessibility of health care, which created more value for cancer patients [11]. At such a crucial juncture, several important questions arise in the minds of patients and clinicians. We checked the related literature and found very few research articles in this context. Therefore, the main aim of this systematic review was to identify the quality of life of people with cancer in the era of the COVID-19 pandemic in India.

## 2 MATERIALS AND METHODS

### 2.1 Study Selection

Studies that were included satisfied the following criteria: (a) Treatment and care of cancer patients in the COVID-19 era and (b) published in the English language. The exclusion criteria include no specific limitations pertaining to the study duration or any study type.

### 2.2 Systematic Search Strategy

The researchers did extensive literature searches on databases, *i.e*., PubMed, Medline, Embase, Clinical Key, and Google Scholar till 3rd Feb 2021. Title and abstract were searched by following terms: (“quality of life” OR “quality” OR “quality of life” OR “persons” OR “people” OR “neoplasms” OR “cancer” OR “COVID-19” OR “covid 19 pandemic” OR “India”) AND (“life” AND “persons” AND “neoplasms” AND “era” AND “COVID-19” AND “India”). All titles and abstracts of articles were screened by the following inclusion criteria by two investigators (KKR + CVK). For articles that fulfill inclusion criteria, their full texts were retrieved. In the case of eligibility of article uncertainty between KKR and CVK, the final decision was taken by SG. Grey literature like Google Scholar was also included. A final study was conducted by the Department of Surgery, AIIMS, Rishikesh, India, on 10th Feb 2021 to find studies using the above methods.

### 2.3 Data Extraction

For each article, data were extracted, including the author's name, year and location of the study, type of study, participants, and area of study. PRISMA data evaluation tools were used to obtain information from each study.

### 2.4 Data Synthesis

No studies were obtained from the Indian subcontinent regarding the quality of life of cancer patients in the COVID-19 era. The data related to cancer patients’ treatments and other aspects of the COVID-19 era in India was obtained and reported.

### 2.5 Quality Assessment

All articles that were selected by two investigators (KKR + CVK) were assessed for validity and authenticity by five experts from different oncology departments.

## 3 RESULTS

### 3.1 Study Characteristics

**Fig. (1):**
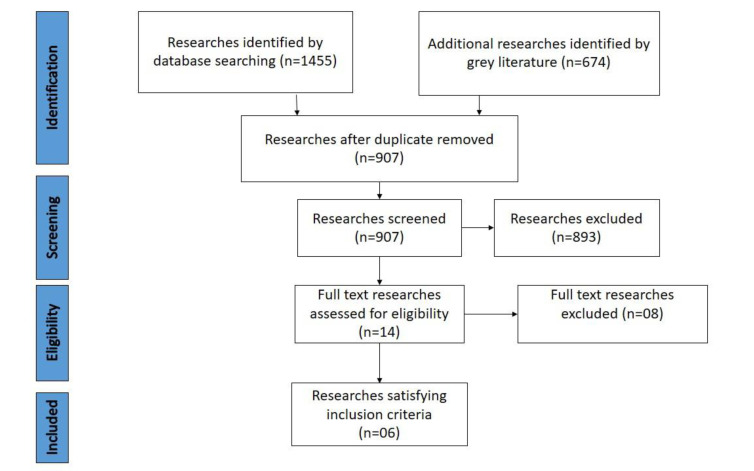
PRISMA flow chart of systematic search [34].

The database search was conducted till 3rd Feb 2021. The database search obtained a total of 1455 articles, including n=233 articles in PubMed, n=221 articles in MedLine, n=225 articles in Embase, and n=102 articles in Clinical Key. Additional records (n=674) were identified through grey literature (Google Scholar). After the removal of duplicate articles, 907 articles were screened (Fig. **1**).

Upon abstract screening, 893 articles were excluded due to unrelated research topics, failing to complete inclusion criteria, or not being published in English. Then, 14 full-text articles were assessed, of which 06 articles qualified for inclusion criteria of the study [12-17] (Table **1**).

### 3.2 General Finding of Included Studies

Table **1** summarizes the eligibility for each study. Most studies were published in 2020. Included studies were conducted by New Delhi (n=4) [12, 15-17], followed by other tertiary cancer care institutions (n=2) [13, 14] in India. The majority of the studies were review articles [13, 16], followed by observational studies [14, 15], editorial articles [12], and guidelines by IAPC [17]. Most of the included studies were conducted on either cancer patients [12, 13, 15, 16], advanced cancer patients [17], or health care workers [14].

#### 3.2.1 Cancer and its Association with COVID-19 Diagnosis

Currently, in India, approximately 4.5 million cancer patients are present, who are either at different stages or in follow-up treatment. Annually, over 1.5 million new cancer patients are registered in India [18]. Patients who suffer from any non-communicable diseases, *i.e*., cancer, diabetes, cardiovascular disease, chronic respiratory or kidney disease, are more vulnerable to death related to COVID-19 illness [19]. Around 85% of COVID-19 infected patients who died had more than one or more comorbidity [20]. A study showed that among cancer patients, there is an increased probability of up to twofold of getting COVID-19 infection and a fourfold increased probability of death than in normal patients [13]. The case fatality rate (CFR) among cancer patients with COVID-19 is 14% [15].

#### 3.2.2 Side-effects of Postponing Cancer Treatment during COVID-19

There are several theories regarding the efficacy of cancer treatment modalities during the COVID-19 outbreak. To date, researchers have not found any evidence to draw conclusions regarding whether cancer treatment is effective or whether it should be postponed further during a pandemic. A study also reported that the majority of cancer patients presenting to hospitals at an advanced stage are not getting appropriate treatment because of COVID-19 and feeling threatened with life [12]. A cohort study found that twenty percent of patients who were positive for COVD-19 also had an associated diagnosis of cancer. Their treatment was postponed for 30 days [21], *i.e*., chemotherapy and surgery did not show any poor results in this study, which again indicated that postponing of cancer treatment within 30 days showed no negative effects on the outcome of the cancer patient. Another study evidence showed that delays in cancer surgery increase the risk of disease progression and further increase the chances of metastatic disease and decrease life expectancy and survival of patients [22]. If cancer surgery is postponed for three months, there is a reduction in average life-years gained (LYG), and resource-adjusted life-years gained (RALYGs) per patient, so prompt action is of utmost importance for any cancer patient [23].

#### 3.2.3 Obstacles during Surgery of Cancer Patient with COVID-19

During the COVID-19 pandemic, regular screening of each patient with RT-PCR with either oral or nasal swabs for COVID-19 was mandatory within 72 hours before any elective surgery [24]. During any emergency surgery, there was an exception, *i.e*., a patient’s life-threatening condition. Research evidence shows that if surgery is postponed for any cancer patient for four weeks, it is further associated with increased mortality and decreased pulmonary complications [13].

Globally, there is an acute shortage of personal protective equipment and N95 respirators, and this problem is worsening in developing countries like India [25]. There was a lack of research on whether the use of PPE showed any significance under this COVID-19 scenario during surgery. The CDC also recommended the use of N95 respirators during high-risk procedures only but did not mention surgical procedures [26]. In developing countries, non-academic institutions or smaller hospitals also have a major issue with insufficient budgets. Therefore, the availability and efficacy of PPE were the main challenges faced by surgeons in India [25]. Another study showed that the non-compliance rate of healthcare workers in operation theatres was 7%, which could be another method of COVID-19 transmission among health care professionals [14].

During the COVID-19 pandemic, various surgical societies offered little guidance and advice for minimally invasive laparoscopic and robotic surgery [27]. All societies point out that there is no significant evidence regarding the avoidance of minimally invasive laparoscopic patient surgery for cancer [28, 29]. It not only provides main benefits but also shortens the length of the hospital stay of the patient, which is very much a critical situation currently.

#### 3.2.4 Obstacles during Chemotherapy and Radiation Therapy

Chemotherapy is another treatment modality used for the treatment of cancer patients. During any chemotherapy, there is always a requirement for logistics and chemotherapeutic drugs. For example, oral chemotherapeutic agents and their proper scheduling should be correlated to minimize exposure and further reduce hospitalization among cancer patients [30]. Prioritization of patients should be properly met, and it must be a joint decision of patients, caregivers, and physicians. Cancer societies must provide guidelines for cancer practitioners during this difficult period to meet the local needs of every cancer patient.

Radiation therapy is also mainline of treatment for cancer. The treatments last for a few weeks, in which each fraction of the treatment is often given for a few minutes. Radiation therapy mainly requires minimal exposure time if appropriate precautions are taken. During the COVID-19 pandemic, hypo-fractionation, advanced prevention, and preparation strategies for radiation therapy have been used to minimize exposure [31]. Cancer societies provided guidelines for the treatment of cancer patients during this difficult period. A study conducted among cervical cancer patients showed that each oncologist followed a “Do No Harm” approach during the treatment of cancer patients. During cancer service delivery, oncologists try to minimize the risk of infection by adopting a holistic approach for the therapy and diagnosis of cancer patients in India [16].

#### 3.2.5 Palliative Care for Cancer Patients as per IAPC Guideline

Palliative care is very much a priority in the case of any pandemic, as palliative patients need supportive care all the time [32]. IAPC provides guidelines for providing palliative care and treatment for advanced cancer patients in the COVID-19 pandemic [17]. Palliative care outreach is also very important now, as people who are suffering from advanced cancer may not be able to reach the hospital and also lack family or financial support [33]. Traveling to the oncology center was also difficult for specialist oncology. Therefore, psychosocial treatment, multi-disciplinary coordination, and proper distribution of resources were required to manage this situation. Other healthcare staff should be further trained and prepared to deal with symptom management, alternative strategies, and bereavement support for patients and family members. In pandemic scenarios, the main focus of palliative care should be early discharge of patients from cancer institutions and if possible, provide palliative care at home or with their loved ones [34].

#### 3.2.6 Measures taken to Combat the COVID-19 Pandemic: Tele-Medicine Consultation

During a pandemic, the government of India started telemedicine consultations in which there were no social contacts, which is a valuable asset for both patients and healthcare professionals [35]. The risk-adapted model was selected for it, *i.e*., patients who were already registered in the hospital and who were on follow-up can easily assess telemedicine consultation [36] in the near future before the pandemic is over.

### 3.3 Non-specific Findings

#### 3.3.1 Number of Oncologists and Cancer Patient Burden in Post-COVID-19 Pandemic Era

Globally, in the pre-COVID-19 situation, cancer units were overworked, with a huge number of cancer patients among developed and developing countries [9]. Due to COVID-19, cancer patients are diverted to an oncology unit. Therefore, in the near future, the service of cancer care facilities will not be able to combat the demand for cancer care. The management of older patients, along with newly diagnosed cancer cases in the coming months, will be a huge challenge. This extremely difficult scenario needs to be addressed on an urgent basis in order to avoid delays in treatment, reduce psychological trauma for cancer patients, ensure the long-term survival of cancer patients, and reduce the burn-out of medical and nursing staff. In addition, countries like India should make a plan for cancer preparedness and a response plan for their cancer patients with COVID-19 positive diagnoses.

#### 3.3.2 Effect of COVID-19 on the Mental Health of Physicians

Similar to cancer patients, physicians are also vulnerable to extreme mental stress during this crisis. This includes extended and exaggerated working hours, lack of breaks, and a constant need to be alert in a high-risk setting. The CDC reported in August 2020 that about one in four hospital employees seriously faced declining mental wellbeing and had suicidal ideation in the last 30 days [37].

#### 3.3.3 Effect of COVID-19 Pandemic on Cancer Patients’ Psychological Wellbeing

During this COVID-19 pandemic phase, cancer patients have suffered from emotional trauma. Research evidence shows that the diagnosis of cancer has a strong association with a profound psychological effect, which changes a patient’s life completely and causes a persistent threat to their survival [38]. Research evidence from China also indicates that COVID-19 triggered a rise in anxiety, stress, insomnia, and depression among patients [39] and their family members. When both deadly diseases combine, they ultimately cause profound psychological effects.

#### 3.3.4 Effect of COVID-19 on Quality of Life of Cancer Patients

Cancer treatment delivery was offered to each cancer patient as per standard safety protocol. The best treatment decisions were made through proper scheduling and setting of priority [40]. However, the overall quality of life of each cancer patient is very much affected [12]. To date, as per literature research, no study has been conducted in India to assess the quality of life of cancer patients. This issue needs to be discussed with health care administrators and policymakers in the country for further interventions as soon as possible.

## 4 DISCUSSION

This COVID-19 pandemic has highlighted our shortcomings in cancer care in India, which has taught us the importance of more cancer centers in India, especially in rural areas. All these measures were to minimize inequalities and disparities in the accessibility of cancer care by the general population. Therefore, the present study greatly supports more cancer treatment facilities in the post-COVID-19 pandemic era.

The COVID-19 pandemic has caused wide gaps and disparities in cancer treatment; therefore, present cancer care facilities cannot fulfill the needs of cancer patients in the future. The general public had high hopes for COVID-19 vaccines and cancer care delivery services. In the post-COVID-19 pandemic era, cancer care will be a major challenge for oncologists. It will help a huge number of cancer patients who have been waiting for treatment for many months. Therefore, for cancer patients who are at risk for delayed cancer therapy, mortality and morbidity increase.

Before and after the COVID-19 pandemic, the government of India must focus on improving cancer care infrastructure and incorporate strategies and evidence from other countries into the management of cancer patients. Even during this pandemic, by following adequate precautions, cancer care was provided to cancer patients.

### 4.1 Limitation

No direct research was found during a literature search for quality of life assessment of cancer patients in the Indian subcontinent. Despite this, studies related to a few areas, *i.e*., cancer and its association with COVID-19, cancer treatment modalities, and palliative care in the COVID-19 era, were overlooked to check cancer care delivery in this present scenario. In the coming future, perhaps a few studies will come up to address issues of the quality of life of cancer patients.

## CONCLUSION

This systematic review demonstrated up-to-date evidence regarding cancer treatment modalities for patients in the COVID-19 era in India. No direct research was identified in PubMed, Medline, Embase, Clinical Key, and Google Scholar databases by investigators to assess the quality of life of cancer patients in the Indian subcontinent, so the quality of life for cancer patients is still unrevealed. Evidence from global studies indicates an altered quality of life for cancer patients in the COVID-19 pandemic. Evidence from research studies showed that cancer physicians followed evidence-based decisions and incorporated multi-disciplinary management into decision-making for cancer patients during the pandemic era. The government should emphasize the role of social media (*via* telemedicine consultation) in creating awareness amongst the general public regarding cancer symptoms during the COVID-19 pandemic.

## Figures and Tables

**Table 1:** Research evidence for cancer patients.

**Author, Year**	**Methodology**	**Participants**	**Ward, Area**	**Research Tools**	**Results**
Abhishek Shankar, 2020, New Delhi [12]	Editorial article	Cancer patients	Department of Radiation Oncology	-	Patients faced threats and delayed cancer treatment.
Mainak Chakraborty, 2020, Varanasi [13]	Review article	Cancer patients	-	-	Results show that among cancer patients, there is a twofold increased probability of getting COVID-19 infection and a fourfold increased chance of death than normal patients.
Gagan Prakash, 2020, Maharashtra [14]	Observational study	Health care workers of cancer patients	Operating room	WHO surgical safety checklist	To reinforce the use of appropriate PPE for cancer patients with COVID-19. -Overall compliance was 96.3%. -Eye protection non-compliance was 7%.
Anurag Mehta, 2020, New Delhi [15]	Observational study	Cancer patients	Emergency department	RTPCR test	The CFR among cancer patients was 14%.
Abhinav Dewan, 2020, New Delhi [16]	Review article	Cervical cancer patient	-	-	- Oncologists should follow the “Do No Harm” approach. -During cancer service delivery, try to minimize the risk of infection. -Adopt a holistic approach for therapeutic and diagnostic purposes.
Anuja Damani, 2020, New Delhi [17]	Guideline for Palliative care	Advanced cancer patient	-	-	Provide guidelines for palliative care in the COVID-19 pandemic.

## LIST OF ABBREVIATIONS

COVID-19: = Coronavirus Disease
IAPC: = Indian Association of Palliative Care
RT-PCR: = Reverse Transcription-Polymerase Chain Reaction
**CDC**: = Centre for Disease Control and Prevention
**WHO**: = World Health Organization
**PRISMA**: = Preferred Reporting Items for Systematic Reviews and Meta-Analyses
